# Association of Polymorphism with Periodontitis and Salivary Levels of Hypoxia-Inducible Factor-1α

**DOI:** 10.1055/s-0044-1785530

**Published:** 2024-05-14

**Authors:** Wael Abdulazeez Kzar, Raghad Fadhil Abbas

**Affiliations:** 1 Department of Periodontology, College of Dentistry, University of Baghdad, Baghdad, Iraq

**Keywords:** genetic, periodontitis, diagnosis, HIF-1α

## Abstract

**Objective** This investigation aims to investigate the association between HIF-1α genetic polymorphism and periodontitis and examine and contrast the levels of HIF-1α present in the saliva of subjects afflicted with periodontitis and in the control group. Additionally, this study aims to establish diagnostic proficiency of this biomarker in distinguishing between periodontal health and disease.

**Materials and Methods** This study entailed the collection of venous blood samples and unstimulated saliva samples from a total of 160 participants, encompassing 80 individuals diagnosed with periodontitis and 80 periodontitis-free individuals. The periodontal parameters were evaluated, involving the measurement of clinical attachment loss, the probing pocket depth, and the bleeding on probing percentage. Subsequently, genetic analysis of HIF-1α using polymerase chain reaction (PCR) technique, DNA sequencing, and enzyme-linked immunosorbent assays was conducted.

**Results** The genetic analysis of 352 bp of the HIF-1α gene revealed the presence of 66 single-nucleotide polymorphisms (SNPs) in control samples, whereas 78 SNPs were found in periodontitis sample. The nucleotide A was replaced with a C nucleotide at position 207 of the amplified PCR fragments. The homozygous AA pattern was predominant in the control group, with significant differences between the two groups. In contrast, the homozygous CC pattern was more dominant in the periodontitis group, with significant differences between the two groups. The analysis of Hardy–Weinberg equilibrium for the comparison between the observed and the expected genotypes showed significant differences between the observed and the expected values in the control and periodontitis groups, as well as the total sample. The highest mean values of the measured periodontal parameters were found in the periodontitis group (clinical attachment loss = 4.759, probing pocket depth = 4.050, and bleeding on probing = 30.950) with statistically significant differences between the groups. The periodontitis group showed significantly higher salivary HIF-1α levels compared to control group ( *p* < 0.001). Besides, HIF-1α is a good biomarker in distinguishing between periodontal health and periodontitis.

**Conclusion** rs1951795 SNP of HIF-1α has no significant impact on the progression of periodontitis and the salivary level HIF-1α. Periodontitis results in a notable elevation in HIF-1α salivary levels, with an outstanding diagnostic ability to distinguish between periodontitis and periodontal health.

## Introduction

Periodontitis is a persistent multifactorial inflammatory disease involving microbial, genetic, and environmental factors. Determining disease initiation and progression depends on the susceptibility of the host and the host's interaction with the microbiota within the biofilm. 1 2 3 Many genetic loci are involved in this process for the onset of periodontitis, which differs among ethnic groups and can be affected by environmental variables. 4 5

Single-nucleotide polymorphism (SNP) is recognized as the most prevalent form of genetic variation, characterized by the replacement of one nucleotide by another. SNPs commonly occur near protein-coding genes and can impact the expression of these genes or the synthesis of proteins. This, in turn, can influence the structural components of the periodontium or the immune response of the host to microbial attacks. Polymorphisms can either improve or hinder the production of certain cytokines and enzymes that primarily influence immune and inflammatory responses such as interleukin-1, interleukin-10, interleukin-1β cyclooxygenase-2, and prostaglandin E2. Moreover, studies have found that genetic disparities among the population are associated with differences in the severity and development of periodontitis. 6 7 8

Maintaining oxygen homeostasis is an essential prerequisite for the viability of multicellular organisms. 9 Hypoxia is a physiological state characterized by a reduction in the availability of molecular oxygen (O2) or is linked to a disruption in the regulation of oxygen levels during intracellular redox reactions. 10 Studies showed that hypoxia has significantly impacted multiple illnesses, most notably in inflammatory disorders such as rheumatoid arthritis, diabetes, ischemic heart disease, inflammatory bowel disease, and cancer. 9 11 12

The biological response of the body to oxygen deficiency is the accumulation and stimulation of hypoxia-inducible factor-1 alpha (HIF-1α), which is a significant nuclear transcription factor present in mammalian cells and regarded as a crucial cellular transcription factor that facilitates the adaptive responses to low oxygen levels in tissues and cells, including the human periodontium. 13 14 15 Furthermore, it has been observed in various studies that the transcriptional function of HIF-1α is increased by the widely recognized pro-inflammatory cytokine tumor necrosis factor-alpha under normal oxygen conditions. This effect is mediated by toll-like receptor four and the receptor activator of nuclear factor-kappa B(RANK). 16 17 18

The activation of HIF-1α leads to the transcription of over 60 genes, including erythropoietin and vascular endothelial growth factor (VEGF). These genes play crucial roles in biological processes such as erythropoiesis and angiogenesis, which contribute to the promotion and enhancement of the delivery of oxygen to hypoxic tissue. 19 20 21

In addition, it has been observed that HIF-1α stimulates the transcription of genes associated with cellular proliferation and viability and regulates glucose and iron metabolism. 21

Studies have observed an increase in HIF-1α protein expression in gingival tissue specimens obtained from patients diagnosed with periodontitis. Ng et al 13 were the first to provide evidence of elevated HIF-1α expression in periodontal tissues affected by periodontitis. Besides, literature documented a significant elevation of HIF-1α expression in the gingival tissue, consistent with the degree of disease severity. 22 23 24

Additionally, the level of HIF-1α in gingival crevicular fluid and saliva is observed to be increased in patients with periodontitis compared to patients with gingivitis or healthy control. 25 The findings mentioned above indicate a potential involvement of HIF-1α in the etiology and progression of periodontal disease.

Presently, clinicians utilize the evidence of radiographic bone loss in addition to the periodontal parameters as a tool of diagnosis to detect and evaluate the extent and the severity of periodontal disease. 26 However, substantial limitations in the utilization of clinical measurements have spurred the investigation of alternate approaches for periodontal disease diagnosis and monitoring. 27

Therefore, the identification of individualized biological indicators such as salivary immune-inflammatory biomarkers that can signal the level of risk, monitor the advancement of the disease, evaluate the health state, and forecast treatment results would offer substantial advantages in therapeutic settings. Saliva is growing in significance as a broadly utilized fluid for diagnostic resolves due to its bioavailability, cost-effectiveness, noninvasive accessibility, limited sensitivity to collection technique, and the comparative stability of salivary specimens upon storage. 28

Considering the adverse consequences of hypoxia on human physiology, 9 11 12 and the crucial involvement of HIF-1α in the development of periodontitis, 22 23 24 25 further studies are needed to recognize the effects of hypoxia associated with periodontitis on the levels of HIF-1α in saliva. Additionally, it is essential to examine the impact of the genetic variation in this factor on its concentration in saliva and the susceptibility of individuals to periodontitis.

This study sought to examine the association between HIF-1α SNP and periodontitis and assess the presence of disparities in HIF-1α levels between persons with periodontitis and those who are healthy. Subsequently, we intended to assess the accuracy of using salivary HIF-1α levels as a diagnostic tool, in comparison to the conventional clinical periodontal measurements, which are the probing pocket depth (PPD) and clinical attachment loss (CAL), to distinguish between periodontal health and periodontitis. To the extent of our current understanding, there is a shortage of prior research examining the potential association between the SNP of HIF-1α and periodontitis and its salivary concentration.

## Materials and Methods

### Study Design

This investigation is a case–control observational study at the College of Dentistry, Baghdad University, Iraq. The study was conducted between February 2022 and October 2022 and involved the voluntary participation of healthy individuals and patients detected with periodontitis. The current investigation adhered to the ethical guidelines outlined in the Declaration of Helsinki by the World Medical Association. Moreover, it obtained approval from the ethics committee in the College of Dentistry / Baghdad University, reference number: 500.

### Study Population

The study sample consisted of 160 Iraqi Arab subjects of both genders with similar ethnic backgrounds. The sample size was calculated based on a prior pilot study to determine the precise sample size for the current investigation, thus avoiding attrition bias. The pilot study took ten samples for both groups, and the enzyme-linked immunosorbent assay (ELISA) examination then achieved to assess the concentration of HIF-1α in saliva. The exact sample size was then calculated according to the formula mentioned by Sharma et al. in 2020 29 for case-control study. “Sample size = r + 1/r × (SD)2 × (Zβ + Zα/2)2/d2”.

Before performing the periodontal examination, both medical and dental records were acquired and thoroughly assessed, taking into consideration the exclusion criteria. Subsequently, the selected participants were categorized into two groups (80 participants for each group) according to their clinical periodontal condition:

- **Control group:** clinically healthy periodontium / systemically healthy individuals.
- **Periodontitis group:** patients with periodontitis / systemically healthy.

The subjects included in this investigation were the control group with healthy periodontium revealing good oral hygiene and had no history or symptoms of periodontal disease with a PPD less than or equal to 3 mm and BOP of less than or equal to 10%, and no CAL. 30 Periodontitis groups were categorized as the existence of interdental CAL at more than or equal to 2 nonadjacent teeth or the presence of buccal or oral CAL more than or equal to 3 mm with the existence of PPD more than 3mm, which can be detected at more than or equal to 2 teeth. All cases in the periodontitis group revealed a generalized form of the disease (≥30% of teeth being affected) and unstable status of the disease (PPD 4 mm with BOP or PPD ≥5mm). 26

### Eligibility Criteria

The patient had to be systemically healthy, except for the specified criteria of the case definition. In addition, the patient should meet the least prerequisite of having at least 20 teeth to be included in this study. Patients also should have no signs of any systemic illness and should not have consumed any antibiotics over the last 3 months. The exclusion criteria included individuals who had medical conditions such as immunologic diseases, hepatitis, and diabetes mellitus, along with those who had received periodontal treatment within the previous 6 months. Individuals who have inflammatory bowel disease (like Crohn's disease), liver or kidney problems, or a history of organ transplants or cancer are also excluded from this investigation.

Additional exclusion criteria encompassed individuals with a history of prior comprehensive periodontal intervention or current active periodontal therapy, and patients with existing periodontal diseases other than periodontitis. Pregnant women, patients who had orthodontic appliances, and patients with oral diseases not related to periodontitis, such as lichen planus and aphthous ulcer, were also not included.

A calibrated examiner conducted the initial examination, which consisted of measurement of the PPD, CAL, and the percentage of bleeding upon probing (BOP).

### Collection of Salivary Sample

Samples of saliva were obtained before the recording of the periodontal parameters. Before the collection of salivary samples, all participants were instructed to abstain from consuming any meal or drink except water for one hour. Then, the participants were asked to sit in a comfortable, relaxed position and allow saliva to flow naturally. A universal saliva collector was employed to collect unstimulated saliva. The patients were instructed to maintain a slightly tilted and forward position of their heads to enhance saliva flow, deprived of any external stimulus, till an amount of 5 mL was achieved. The gathered samples underwent centrifugation for 30 minutes at a speed of 4,500 revolutions per minute. Subsequently, they were preserved at a temperature of −20 °C until further examination.

### Enzyme-Linked Immunosorbent Assays

The samples were defrosted and allowed to equilibrate at room temperature for a few minutes. The amounts of HIF-1α protein in saliva were measured using human HIF-1α ELISA kits from (YL Biont, China) that were based on the Biotin double antibody sandwich technology. The procedure was performed following the instructions provided by the maker of the kit. An optical density (OD) measurement was performed using a microtiter plate reader form (HumanReader HS, manufactured by HUMAN Society for Biochemica and Diagnostica mbH). The OD data were exported to spreadsheets and transformed into concentrations using a biomarker-specific linear regression approach.

### Periodontal Parameters and Clinical Examination

A calibrated examiner documented the periodontal charting of each participant, which included measurements of BOP, PPD, and CAL. The clinical periodontal data was collected by evaluating six locations per tooth (mesiobuccal, buccal, distobuccal, mesiolingual, lingual, and distolingual), excluding wisdom teeth, using a periodontal probe (UNC-15). For recording the BOP score, the probe was gently inserted into the sulcus/pocket until minimum resistance was detected. The probing force was approximately 20 to 25g. Starting with the distal surface of the right upper seven, surfaces that exhibited bleeding within 10 seconds were assigned a score of 1, whereas surfaces that did not exhibit bleeding were assigned a score of 0. Simultaneous measurements of PPD and CAL were conducted. PPD was assessed via measuring the distance from the gingival edge to the base of the pocket. At the same time, CAL was measured in millimeters from the cementoenamel junction to the bottom of the pocket. The diagnosis of periodontitis cases was made based on the clinical examination in this step.

#### Collection of Blood Samples

With 70% isopropyl alcohol, an aseptic venipuncture with a 5-mL disposable syringe and a 20-gauge needle was achieved for each participant to obtain a blood volume of 2 mL from the antecubital fossa. The blood samples were then transferred into (1.5 mg/mL) ethylenediaminetetraacetic acid tube and kept at −20°C for subsequent HIF-1α genetic analysis.

##### DNA Extraction

The procedures involved the extraction of DNA, polymerase chain reaction (PCR) amplification, DNA sequencing process, and data processing. The genomic DNA was extracted from blood samples using the Geneaid extraction kit (Taiwan) according to the provided instructions. The components of the kit include GST Buffer, GSB Buffer, W1 Buffer, Wash Buffer, Elution Buffer (10 mM Tris-HCl, pH8.5 at 25°C), Proteinase K, GS Columns, and 2 ml Collection Tubes. The obtained DNA was then quantified using a Quantus Fluorometer (Promega, United States).

##### Polymorphism Detection

The initial phase of PCR amplification comprised the preparation and optimization of the primers. The primers utilized in this investigation to detect the (HIF-1α) gene were designed by PrimerQuest, a tool provided by Integrated DNA Technology. The prepared primers encompassed the vast of the gene to identify any possible variations that may existed in the study groups and to contrast them to the standard sequences. The Bioneer Company supplied the lyophilized form of these primers: a forward primer (F5-TTCCATGTCCTTTGTGTCCAG-3) and a reverse primer (R5-TGTGGCCTAAGCCATCAACG-3), which produced a 352-base pair (bp) product at an annealing temperature of 60°C.

The primer stock solution was prepared by diluting lyophilized primers in nuclease-free water (NFW) to a concentration of 100 pmol/μL for both the forward and reverse tube and subsequently vortexed. Then, the working solution of the primer comprising 10 pmol/μL was obtained by adding a volume of 90 µL of NFW for forward and reverse primer and 10 µL of stock solution in a 1.5 mL tube, kept in the freezer at 40°C.

For the assessment of the optimal annealing temperature for primers, replication of the DNA sample was done with the same primers at treatment temperatures of 55, 58, 60, 63, and 65°C. Then, the process of PCR amplification was accomplished. The PCR procedure was carried out in a total volume of 20 μL, 5 µL of DNA templet, 1 μL of forward and reverse primers, 12.5 µL of master mix (containing top DNA polymerase, deoxyribonucleoside triphosphates, reaction buffer, tracking dye, and patented stabilizer), and 20.5 µL of distal water using the following scheme, denatured at 94°C/ 10 minutes, followed by 30 cycles of denaturation at 94°C/35 seconds, then annealing at 60°C/ 30 seconds, and extension at 72°C/35 seconds. Following a final extension incubation at 72°C/10 minutes, the procedures were stopped by achieving an incubation for 10 minutes at 4°C.

To confirm the presence of amplification following PCR amplification, 0.8% of Agarose gel electrophoresis (Bioneer, South Korea) accompanied with 10 mg/mL ethidium bromide staining (EBS) (Biobasic, Canada) was used. Afterward, 5 μL of PCR cycling product was added to the well directly; an electricity of 100 volts and 50 amperes and was turned on for 1 hour. DNA migrates from the cathode pole to the anode pole. The gel was observed for EBS bands under ultraviolet light, and the result was photographed using a mobile phone camera. The PCR amplicons were sent for Sanger sequencing to Macrogen Company, Korea, utilizing the automated DNA sequences (ABI3730XL) next to the appearance of the PCR amplifies gene fragments with the predicted size to detect the sequences of the nucleotide in these fragments. Only chromatographs that were unambiguous and free from artifacts were subjected to further analysis to ensure the accuracy of the annotation and variations. The virtual position and other specific features of the obtained PCR fragments were identified by contrasting the observed local samples' DNA sequences with the retrieved DNA sequences.

### Interpretation of Sequencing Data

The sequencing results were received by email. The edition, alignment, analysis, and comparison of the received sequences with the corresponding sequences in the reference database were made by using BioEdit Sequence Alignment Editor Software Version 7.1 (DNASTAR, Madison, Wisconsin, United States) and Mega 11. The detected differences in each sequenced sample were then numbered in PCR amplicons and corresponding positions within the reference genome.

### Checking the rs1951795 A207C SNP of HIF-1α

The submission of the detected SNPs to the dbSNP database was made for originality verification. Each specific SNP was highlighted based on its position within the reference genome. Consequently, the detection of the existence of the previous SNP was done by observing its corresponding dbSNP position. Then, the dbSNP position for the detected SNP was documented.

#### Statistical Analysis

GraphPad Prism (v. 9.0) software was utilized to describe, analyze, and present data. Results were presented in terms of mean, percentage, and standard deviation. Shapiro–Wilk normality test was done to check the data distribution. An unpaired t-test was used to achieve the comparison between the two groups. For the determination of the sensitivity and specificity of the biomarker, the receiver operating characteristic curve, together with the area under the curve (AUC), was utilized. The odds ratio quantifies the degree of association between the occurrence of specific genetic variations and the disease condition. For the calculation of the expected alleles from the observed genotypes, Hardy–Weinberg equilibrium was used. Multiple linear regression tests analyzed the association between the clinical parameters and HIF-1α gene polymorphisms. Statistical analysis was considered significant when the *p* -values were below 0.05. To prevent bias, the statistical analysis and interpretation of biomarker data were conducted by an investigator who was unaware of the clinical measurement results and the group assignment.

## Results

### Characteristics of Study Populations

A total of 260 subjects were examined to test their eligibility for inclusion criteria in the current investigation; about 100 were excepted for various reasons, including the presence of systemic diseases such as diabetes, pregnancy, or declination to participate. Consequently, 160 volunteers (146 males and 14 females with an age range (28-61) participated in the current study and were separated into two groups. As illustrated in Table 1 , it shows the frequency distribution of the participants by mean participant age and mean clinical periodontal characteristics in each group.

**Table 1:** Age and clinical periodontal parameters in healthy control and patients with periodontitis

	Control		Periodontitis		
	Mean	± SD	Mean	± SD	*p* -Value
Age	31.500	2.028	40.955	10.750	0.078 ns
BOP	6.350	1.493	30.950	14.525	0.000 a
PPD	2.34	1.02	4.050	0.016	0.000 a
CAL	0.00	0.00	4.759	0.152	0.000 a

All results in the table were obtained from the *t* -test: BOP, bleeding on probing; CAL, clinical attachment loss; ns, not specified; PPD, probing pocket depth; SD, standard deviations; level of significance as not significant *p* > 0.05.

a Significant at *p* < 0.001.

### Genetic Analysis

**Fig. 1:**
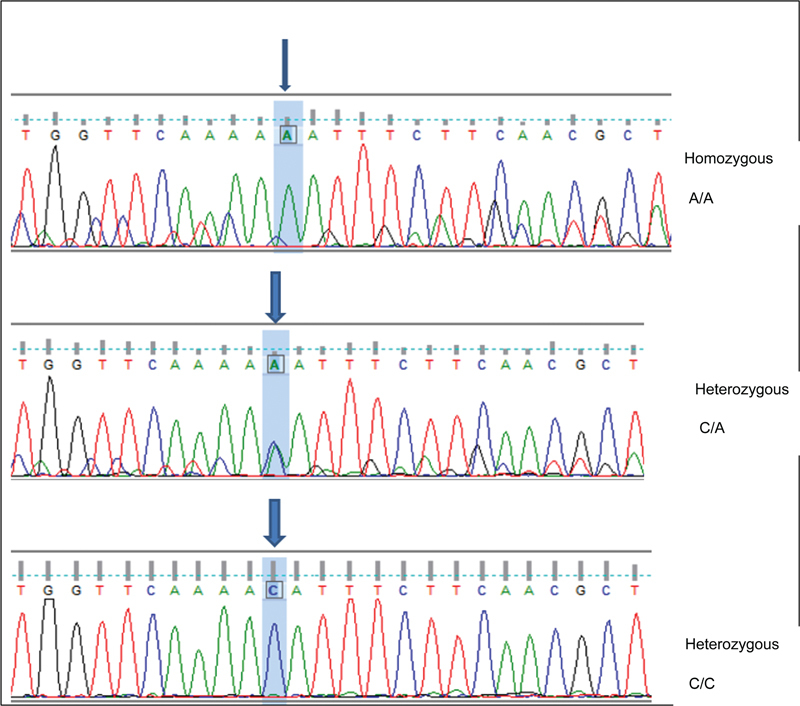
The pattern of the detected single-nucleotide polymorphisms (SNPs) within the DNA chromatogram of the targeted 352 bp amplicons of the hypoxia-inducible factor-1α gene (Sanger sequencing). The identified SNPs were highlighted according to their positions in the polymerase chain reaction amplicons.

The reactions of sequencing specified the particular identity after achieving NCBI blastn for PCR amplicons ( https://blast.ncbi.nlm.nih.gov/Blast.cgi ). Regarding the supposed 352bp amplicons, the NCBI BLASTn engine showed sequence similarities of about 99% between the sequenced samples and the reference target sequences. The sequencing chromatogram files of the detected single nucleotide variation and detailed annotations were documented and shown according to its corresponding locus in the amplified 352 bp PCR fragments ( Fig. 1 ).

Regarding the presence of the SNP in the samples, the nucleotide A was replaced with C nucleotide in position 207 of the amplified PCR fragments, namely 207 A > C, or A207C at rs1951795. The allele frequency analysis showed a significant increase in the C allele compared with the A allele in both groups at a *p* -value of 0.0001 with an odd ratio equal to 0.2. However, the sequencing results of the 352 bp of the HIF-1α gene revealed the presence of 144 SNPs in total (66 SNPs in control samples, whereas 78 SNPs were found in periodontitis sample). The A allele was noticed with increased frequency in the control versus periodontitis group. Consequently, the homozygous AA pattern was identified in only two periodontitis samples (2/80), while it was significantly higher in the control samples (14/ 80). At the same time, the heterozygous AC pattern was observed in 5 control samples (5/80) and three periodontitis samples (3/80). Remarkably, the homozygous CC pattern was detected in 75 of the periodontitis samples (75/80) and 61 of the control samples (61/ 80), with a significant odd ratio as illustrated in Table 2 .

**Table 2:** Allele and genotype frequency of HIF-1α (207 A > C) SNP among periodontitis and control

Alleles	Periodontitis: 80	Control: 80	OR	*p* -Value
A	7	33	0.2	0.0001
C	78	66		
Genotype				
AA	2(2.5)	14(17.5%)	0.1	0.006
AC	3(3.75)	5(6.25)	0.6	0.5
CC	75(93.25)	61(76.25)	5	0.004

Abbreviations: A, adenine; C, cytosine; HIF-1α, hypoxia-inducible factor-1α; OR, odds ratio; SNP, single-nucleotide polymorphism.

The analysis of Hardy–Weinberg equilibrium showed significant differences between the observed and the expected value in control and periodontitis groups, as well as the total sample, as illustrated in Table 3 .

**Table 3:** HWE analysis for control, periodontitis, and total samples

HIF-1α	Control		Periodontitis		Total samples	
Genotypes	Observed	Expected	Observed	Expected	Observed	Expected
AA	14	3.4	2	0.2	16	0.2
AC	5	26.2	3	6.7	8	6.7
CC	61	50.4	75	73.2	136	73.2
HWE	52.373		24.360		95.21632653	
p-Value	0.000		0.000		0.000	

Abbreviations: A, adenine; C, cytosine; HWE, Hardy–Weinberg equilibrium; HIF-1α, hypoxia-inducible factor 1-alpha.

### Immunological Analysis

**Fig. 2:**
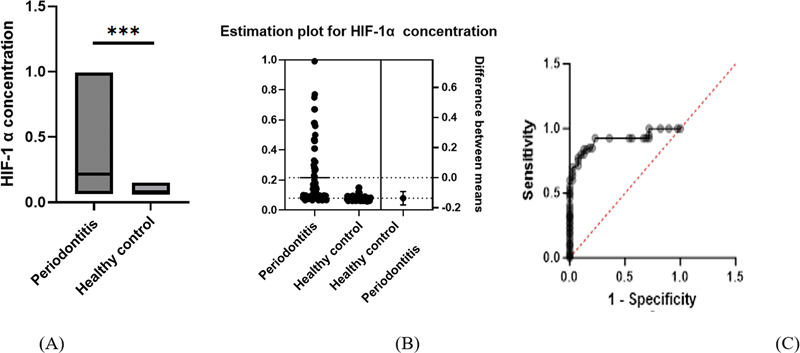
Comparisons of salivary biomarkers among groups ( **A** ) salivary concentration of hypoxia-inducible factor (HIF) between groups; ( **B** ) *t* -test plot for HIF concentration between groups; ( **C** ) receiver operating characteristic curve for salivary HIF-1α concentration; *** significant at *p* -value less than 0.01; presentation done with floating chat where the lower border represents the minimum, the upper border represents maximum, while the internal line inside the chat represents the mean of salivary biomarkers involved in the study.

Concerning the salivary level of HIF-1α, the significantly highest value was found in the periodontitis group. Furthermore, the result of the diagnostic accuracy of the biomarker to discriminate periodontal health from periodontitis was illustrated in Table 4 and Fig. 2 . The AUC for salivary HIF-1α was 0.913, with an excellent ability to differentiate periodontal health from periodontitis.

**Table 4:** Diagnostic accuracy and salivary levels of HIF-1α in ng/mL in the study group

Characteristic	Mean ± SD (ng/mL)	AUC	*p* -Value	Confidence interval	Cutoff point
Periodontitis	0.216 ± 0.02	0.913	0.0001	0.862–0.993	0.08
Control	0.079 ± 0.01				

Statistical analysis between means was done using a *t* -test. AUC, area under the curve; HIF-1α, hypoxia-inducible factor 1-alpha; SD, standard deviation.

### Multiple Linear Regression Analysis

Multiple linear regression was conducted on study samples with different models; in the first model, where CAL is considered as the dependent variable representing a continuous variable for the disease progression in periodontitis, the results showed a significant association between the CAL and salivary level of HIF-1α as well as PPD and BOP. In contrast, there was a nonsignificant association between CAL and genotypes AA, AC, and CC. Additionally, the second model, where the salivary HIF-1α is the dependent variable, seeks the effect of SNP and periodontal parameters on salivary HIF-1α. The result showed a significant association between salivary HIF-1α and CAL, BOP, whereas no significant association was found between PPD and the SNPs, as presented in Table 5 .

**Table 5:** Multiple linear regression analysis

Variable	Estimate	SE	95% CI	| *t* -Test|	*p* -Value	
First model (CAL is the dependent variable)						
Intercept	−1.714	0.259	−2.227–1.201	6.600	<0.000	a
Age	−0.003	0.003	−0.010–0.004	0.8565	0.393	ns
PPD	0.582	0.061	0.461–0.704	9.476	<0.000	a
BOP	0.020	0.007	0.005–0.036	2.641	0.009	b
HIF-1α	0.923	0.291	0.348–1.499	3.172	0.001	b
Genotype AA	0.001	0.140	−0.276–0.279	0.010	0.990	ns
SNP AC + CC	−0.114	0.189	−0.488–0.259	0.604	0.546	ns
Periodontitis	3.564	0.218	3.133–3.995	16.34	<0.0001	a
Second model (salivary HIF-1α is the dependent variable)						
Intercept	0.114	0.079	−0.042–0.271	1.439	0.1521	ns
Age	0.001	0.001	−0.001–0.002	0.2591	0.7959	ns
PPD	−0.020	0.021	−0.062–0.021	0.9716	0.3328	ns
CAL	0.068	0.021	0.025–0.110	3.172	0.0018	b
BOP	−0.006	0.002	−0.010–0.002	3.172	0.0018	b
Periodontitis	−0.056	0.098	−0.251–0.138	0.5756	0.5657	ns
Genotype AA	0.03277	0.038	−0.042–0.108	0.8613	0.3904	ns
SNP AC, CC	0.02135	0.051	−0.080–0.122	0.4155	0.6784	ns

Abbreviations: A, adenine; BOP, bleeding on probing; C, cytosine; CAL, clinical attachment loss; CI, confidence interval; HIF-1α, hypoxia-inducible factor-1α; ns, nonsignificant at *p* -value ≥0.05; PPD, probing pocket depth; SE, standard error; SNP, single-nucleotide polymorphism.

a Significant at *p* -value < 0.0001.

b Significant at *p* -value < 0.01.

## Discussion

The progression of periodontitis, a complicated illness, is influenced by various factors, including genetics. 5 31 The association between the SNPs of the inflammatory mediators and cytokines and periodontitis has gained significant interest in recent research as potential contributors to periodontal disease. 32 33 These investigations can provide a more precise understanding of the development and progression of periodontitis and contribute to the development of novel treatment and preventive approaches. 34

Periodontitis has been linked to hypoxia, which is a recognized risk factor, and studies documented a marked consequent elevation in the HIF-1α expression in saliva, gingival crevicular fluid, periodontal ligament cells, and gingival tissue adjacent to the periodontal pocket. 13 22 23 24 25 Moreover, there is growing evidence of its correlation with abundant inflammatory biomarkers. Furthermore, the validation of a periodontal disease diagnosis necessitates dental knowledge, notwithstanding the limits of clinical periodontal indicators in diagnosing the disease during its initial phase. On the other hand, it should be feasible for other professionals and patients themselves to identify and monitor diseases. Consequently, the purpose of the present research was to investigate the association between HIF-1α SNP and periodontitis and to search for the diagnostic accuracy of the salivary level of this biomarker by analyzing its reported sensitivity and specificity regarding the clinical periodontal measurements of periodontitis patients as saliva is a crucial diagnostic fluid because of its bioavailability and non-invasive accessibility.

Regarding the genetic analysis of the study samples, the homozygous AA pattern was significantly higher in the control group than periodontitis group. Yet, the results of multiple linear regression revealed a nonsignificant association of A207C SNP with periodontitis as well as with salivary levels of HIF-1α level. So far, the credibility of the study above results is difficult to generalize as it was the first study to investigate the association of this SNP with periodontitis conducted on a sample of the Iraqi Arab population. Still, the level of salivary HIF-1α was significantly higher in the periodontitis group than in the control group, with excellent diagnostic ability to distinguish between periodontitis and periodontal health. Studies revealed that periodontal inflammation can exaggerate local hypoxia within the deep periodontal pockets, which leads to activation and maintenance of HIF-1α stabilization during the disease. In other words, hypoxia and HIF-1α increase as an outcome of the heightened inflammatory response and the deeper periodontal pockets, which is crucial for initiation of the reparative mechanism. This mechanism comprises the infiltration of inflammatory cells into hypoxic tissue, the production of cytokines to sustain the inflammatory response, and the activation of angiogenesis and cellular metabolism. 9 Besides, it has been demonstrated that inflammation and hypoxia can mutually influence each other, 9 35 and oxygen metabolism is crucial in maintaining normal physiological functions in periodontal tissues. 36 The fact that hypoxia arises during periodontitis, as well as the subsequent marked elevation in HIF-1α expression and their accompanying role as a pathogenic factor during the disease, is well documented in the literature. Hypoxia may trigger oxidative stress and lead to exacerbated periodontal tissue inflammation. Moreover, hypoxia can enhance osteoclast cell differentiation and bone resorption. HIF-1 also upregulates the transcription of VEGF in inflamed periodontal tissue, which is vastly associated with the probability of bleeding in inflamed periodontal pockets. 13 18 23 25 37 38 39 Since the metabolites of multiplying Gram-negative anaerobic pathogens in periodontal pockets and the impaired microcirculatory perfusion due to endothelial damage and tissue edema in inflamed tissues could be responsible for the development of hypoxic gradient in the vicinity of the periodontal tissue as documented by numerous studies. 40 41 42 43 44 45 Moreover, the proliferation and aggregation of a substantial quantity of inflammatory cells within inflamed periodontal tissue may result in a subsequent decrease in oxygen levels, as these cells rely on oxygen for proper functioning. 43 46

On the other hand, studies revealed the impact of lipopolysaccharide (LPS) released from *Porphyromonas gingivalis (P. gingivalis)* on the level of HIF-1α gene expression. It has been advocated that both LPS and the inflammatory cytokines can induce NF- *κ* B dependent expression of the HIF-1α gene. 47 48 Besides, hypoxia and *P. gingivalis* are claimed to induce HIF-1α and NF- *κ* B activation in periodontal ligament cells synergistically. 22

The current investigation suggested that this hypoxic environment increased HIF-1α level as an adaptive response to oxygen insufficiency in inflamed periodontal tissue. This finding follows various studies that documented a marked elevation in the HIF-1α protein expression adjacent to the periodontal pocket. 13 22 23 24 25

The significant positive association found between HIF-1α level and the parameters BOP, PPD, and CAL in this study could be attributed to the significant immunomodulatory effects of hypoxia on the response of host cells, which disrupt the integrity of the periodontal tissue during the disease, as supported by existing literature.

Hypoxia in the periodontal tissues can be a potent immunomodulatory signal that may upregulate proinflammatory cytokines and matrix metalloproteinase (MMP) expression from host cells, leading to periodontal tissue destruction. 49 50 Hypoxic cells in the periodontal ligament express various mediators, including prostaglandin E2, interleukin-1β, interleukine-6, RANK, and osteoprotegerin (OPG), which affect the expression of receptor activators of nuclear factor-kappa B ligand (RANKL) in the periodontal ligament cells increasing the RANKL/ OPG ratio, a critical pathogenic event in alveolar bone resorption, 38 51 52 which is implicated in periodontal tissue breakdown. 53 54 Besides, hypoxia has been observed to promote LPS-stimulated osteoclastogenesis of bone marrow macrophages and bone resorption. 55

Moreover, it has been observed that hypoxia has the potential to increase the expression of MMP-2 messenger RNA (mRNA) while simultaneously decreasing the expression of tissue inhibitor of metalloproteinase 2 (TIMP-2) mRNA. The modification in gene expression results in a disruption in the proportion of MMP-2 to TIMP-2 mRNA expression. The observed phenomenon exhibits a noteworthy association with the pathophysiology of periodontal disease and assumes a crucial function in the progression of periodontal tissue degradation in individuals with periodontitis. 56

One notable aspect of this study acknowledged as a significant strength is the utilization of salivary biomarkers for diagnosing periodontitis, irrespective of the existence or absence of substantial risk factors such as smoking. This consideration is vital due to the potential for systemic changes in individuals with common risk factors and high-risk medical conditions such as cardiovascular diseases, rheumatic arthritis, and diabetes, 57 58 59 which might undermine the diagnostic efficiency of salivary biomarkers.

In addition, utilizing a predetermined sample, conducting the examination by a calibrated periodontist, and implementing blindness protocols for both the ELISA investigator and statistician collectively mitigate potential biases in the research process. These measures enhance the reliability and validity of the current biomarker's diagnostic capabilities.

Nevertheless, this study investigated the salivary concentrations of HIF-1α in patients diagnosed with periodontitis, irrespective of the degree of disease severity and extent (i.e., the stage and grade of the disease). This consideration is crucial as prior research has established a significant correlation between the degree of inflammation severity in periodontal tissue and the rise of HIF-1α levels, which may potentially influence the outcomes of the present study. 22 23 24 25 Given the potential impact of smoking on oxygen homeostasis in the tissue, additional investigations are essential to determine its effects on the level of HIF-1α.

## Conclusion

rs1951795 A207C SNP at HIF-1α had no role in periodontitis severity and progression as well as on biomarker level associated with this gene. Nevertheless, this study found elevated levels of salivary HIF-1α with excellent diagnostic ability for periodontitis regardless of the existence of a significant risk factor, which is smoking. A significant association between salivary HIF-1α levels and clinical periodontal parameters confirmed this result.
